# The Anti-Colon Cancer Effects of Essential Oil of *Curcuma phaeocaulis* Through Tumour Vessel Normalisation

**DOI:** 10.3389/fonc.2021.728464

**Published:** 2021-10-26

**Authors:** Yewen Feng, Lu Deng, Hengrui Guo, Yumin Zhao, Fu Peng, Gang Wang, Chenghao Yu

**Affiliations:** ^1^ State Key Laboratory of Southwestern Chinese Medicine Resources, School of Basic Medicine, Chengdu University of Traditional Chinese Medicine, Chengdu, China; ^2^ National Engineering Research Center for Biomaterials, Sichuan University, Chengdu, China; ^3^ Southwest Jiaotong University, Chengdu, China; ^4^ West China School of Pharmacy, Sichuan University, Chengdu, China

**Keywords:** *Curcumae rhizoma*, tumour vessel, normalisation, colon cancer, vessel structure

## Abstract

**Background:**

Normalising tumour vessels had become a significant research focus in tumour treatment research in recent years. *Curcumae rhizoma* (CR) is an essential plant in traditional Chinese medicine as it promotes blood circulation and removes blood stasis. Similarly, CR improves local blood circulation.

**Purpose:**

We explored the anti-colon cancer effects of essential oil from CR (OCR) by investigating its role in normalising tumour vessels. We also provided a basis for research and development into new anti-cancer drugs.

**Methods:**

We used colon cancer as a research focus to investigate OCR. We established an *in vitro* co-culture model of colon cancer cells and human umbilical vein endothelial cells (HUVEC). We also established an *in vivo* subcutaneous implant colon cancer model in nude mice. These studies allowed us to evaluate the comprehensive effects of OCR in *in vivo* and *in vitro* colon cancer and its role in normalising tumour blood vessels.

**Results:**

*In vitro*, we found that OCR inhibited Human colon cancer cells (HCT116) and HUVEC cell proliferation and inhibited vascular endothelial growth factor-a (VEGFa) mRNA and protein expression in HUVECs in a co-culture system. Our *in vivo* studies showed that OCR inhibited colon cancer tumour growth, reduced angiogenesis in tumours and increased vascular endothelial (VE)-cadherin and pericyte coverage in tumour vessels.

**Conclusions:**

OCR inhibited colon cancer growth both in *in vivo* and *in vitro* models, reduced angiogenesis in tumours, improved tumour vessel structures and normalised tumour vessels.

## Background

Angiogenesis is an important physiological process during embryonic development, growth and tissue repair. Imbalanced angiogenesis can lead to various pathological consequences and is a particular hallmark feature of tumours (1). From an anatomical perspective, tumour blood vessels are generally disorderly; their generation and distribution are not controlled by normal physiology, leading to spatial abnormalities in blood flow. At the cellular level, the vascular endothelium and pericytes in tumours are missing and cause serious leakage of vascular contents (2–4). This alteration exacerbates local hypoxia and acidosis in tumour environments, eventually converting tumour cells to malignant phenotypes and affecting the killing capacity of immune cells and therapeutics toward tumours (5).

For these reasons, studies have focused on tumour vessel biology. In 1971, researchers pioneered starving tumours to death (6), where tumour formation was inhibited by inhibiting tumour angiogenesis. However, over-pruning tumour vessels had the same effect as blood vessels’ overgrowth. Tumour vessels cannot achieve their normal vascular functions (7). After this, scientists turned their attention to normalising tumour vessels. By regulating the balance of pro- and anti-angiogenic factors, tumour vessel functions and structures could be normalised (8). Recent studies showed that particular drugs could reduce tumour metastases, enhance chemotherapeutic drug delivery and increase the anti-cancer effects of chemotherapeutic drugs after effective vascular normalisation (9–12).

In recent years, bioactive compounds from traditional Chinese medicine and associated derivatives have gained scientific acceptance as potentially promising complementary and alternative medicines to treat various diseases (13–16).

Curcumae rhizoma (CR) is the dried rhizome of Curcuma phaeocaulis Valeton, Curcuma kwangsiensis S. G. Lee et C. F. Liang or Curcuma wenyujin Y. H. Chen et C. Ling plant (13). Traditional Chinese medicine proposes that local tumours suffer from stagnation of ‘qi’, blood and poor metabolism (17). Therefore, for tumour treatment, drugs that promote blood circulation and remove blood stasis are combined to improve local blood circulation. CR is an important traditional Chinese plant that promotes blood circulation and removes blood stasis.

The essential oil from CR (OCR) is the main medicinal active ingredient. The main components are ketones, olefins and aromatic hydrocarbons. Of these, furanodiene, furanodienone, curcumol, β-elemene, curedione and gemmazone are more abundant (17, 18). Studies have shown that CR reduces blood viscosity and improves blood flow rates (19). In addition, evidence suggests that β-elemene increases (VE)-cadherin expression in tumour tissues (20). For these reasons, it is hypothesised that OCR normalises blood vessels in colon cancer tumours.

In this study, we examined OCR’s effects on cell viability, proliferation and vascular endothelial growth factor-a (VEGFa) expression in cultured cell lines human colon cancer cells (HCT116) and human umbilical vein endothelial cells (HUVEC)) *in vitro*. In addition, we simulated HUVEC growth in a tumour environment *in vitro* to study the effects of OCR on the expression of key angiogenic factors. Then, the inhibitory effects of OCR on colon cancer and its effects on tumour vessels were studied *in vivo*. We aimed to explore the promotion of blood circulation to remove blood stasis and provide a basis for developing potential new tumour drugs for colon cancer.

## Methods

### Materials

Cells: HUVECs and HCT116 cells were obtained from the National Engineering Research Centre for Biomaterials of Sichuan University.

Animals: Four-week-old male BALB/C-NU mice were purchased from Sibefu Biotechnology Co., Ltd. (Experimental Animal Licence: SCXK (Beijing) 2019-0010). Nude mice were adaptively fed for one week. Studies were conducted per protocols approved by the Animal Ethics Committee of Chengdu University of Traditional Chinese Medicine (Animal Ethics Approval Number: 2017-08).

Drugs: OCR was diluted in normal saline (Chroma, Chengdu, China). The daily dose was 0.1 mg/g. Oxaliplatin (Solarbio, Beijing, China) was diluted in a 5% glucose solution.

### Cell Culture

HCT116 cells and HUVECs were cultured in RPMI-1640 (Gibco, USA) complete medium containing 10% foetal bovine serum (Gibco) at 37°C in a 5% CO_2_ environment. The indirect co-cultivation system method is adopted to simulate the tumor environment. HCT116 and HUVEC were seeded on the upper or lower layers of the transwell chamber, and the two kinds of cells were placed in the same culture environment. After 24 h of different drug intervention, characterisation studies were performed.

### Animal Studies

*In vivo* studies: Animals were adaptively fed for one week, after which a colon cancer subcutaneous transplantation model was prepared. HCT116 cells were prepared at 1.5 × 10^6^ cells/100 μl normal saline. A 1 ml injection needle was used to subcutaneously inject 100 μl cell suspension into the armpit of mice. Tumour size was measured using a Vernier calliper every three days and tumour volumes calculated according to the formula; V = 1/2 × length × width^2^.

**Figure 1:**
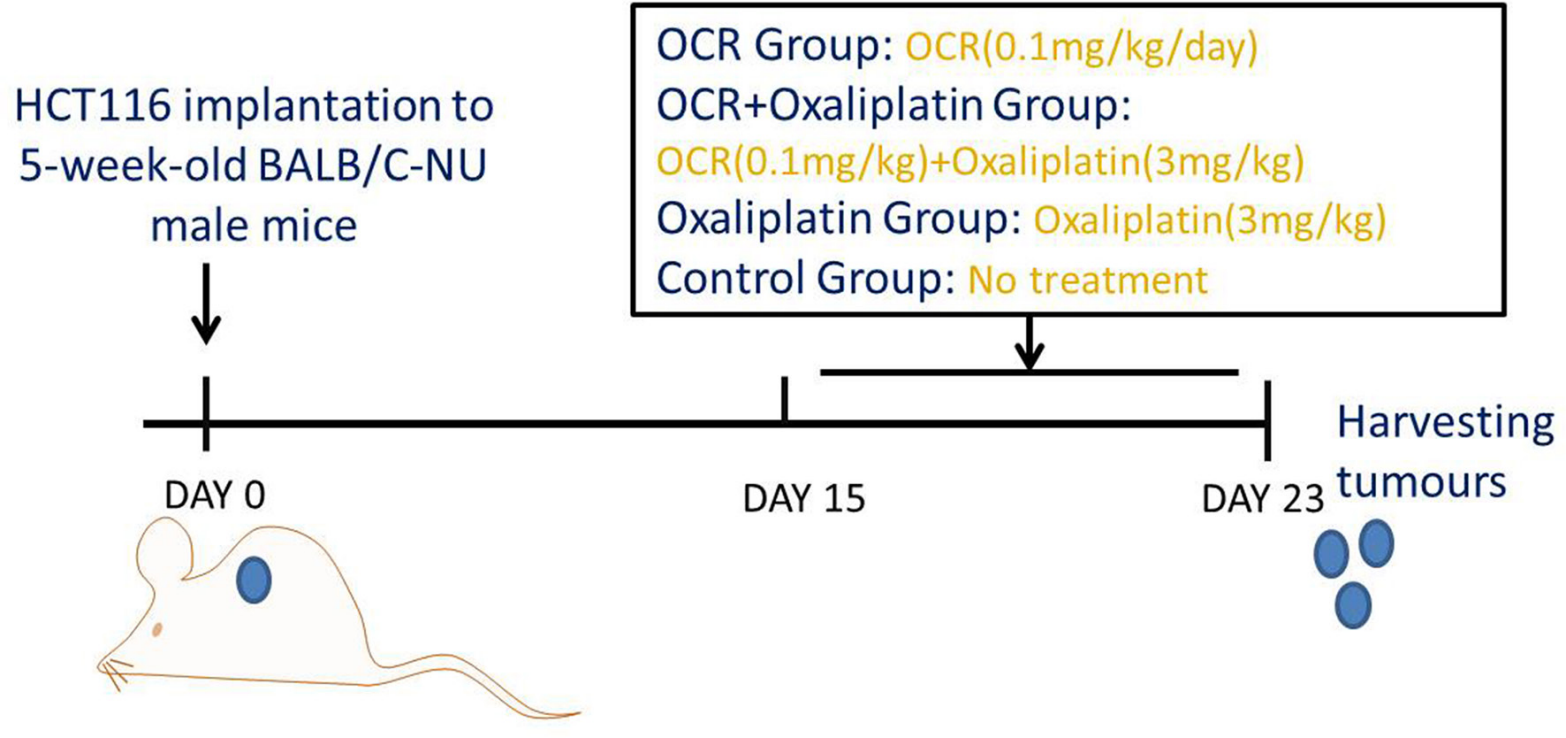
Animal study design.

When the average tumour volume was > 100 mm^3^, we randomly divided the mice into four groups; OCR, OCR + oxaliplatin, oxaliplatin only and model groups. Mice in OCR and OCR + oxaliplatin groups received daily OCR intragastric doses (**Figure 1**). Mice in OCR + oxaliplatin and oxaliplatin groups received intraperitoneal injections with oxaliplatin every 3 days. The model group received no treatment. After study completion, the mice were anesthetised with 2% sodium pentobarbital (0.25 ml/100 g) and sacrificed by cervical dislocation, and subcutaneous tumours removed for immunofluorescence.

### CCK-8 Assay

Cells were seeded in 96-well plates at appropriate densities. After drug intervention, a complete medium containing 10% CCK-8 (Meilunbio, Dalian, China) was added to well for 1 h in the dark. Optical density values at 450 nm were measured using a microplate reader. Cell viability was then calculated.

### The 5-Ethynyl-2´-Deoxyuridine Assay

Cells were seeded in 6-well plates at appropriate densities. After a 24 h drug treatment, an EdU kit (Meilunbio) was used for staining. Cells were observed under a microscope (green fluorescence\ blue fluorescence).

### Quantitative Polymerase Chain Reaction

After extracting RNA, reverse transcription was performed using the HiScript III RT SuperMix kit (Vazyme, Nanjing, China). Then enzyme (Vazyme) was added and PCR instrumentation used (QuantStudio 3, Thermo) for amplification.

### Western Blotting

After lysing cells, lysates were centrifuged and supernatants assayed for protein determination using the bicinchoninic acid (BCA) protein quantification kit (Thermo Fisher Scientific). VEGFa expression (Abcam) was detected, and β-actin (Proteintech, Wuhan, China) used as a loading control.

### Immunofluorescence

Fresh tumour tissue was embedded in paraffin and sectioned. Sections were subjected to antigen retrieval and incubated with CD31 (Servicebio, Wuhan, China), anti- platelet-derived growth factor receptor-β (PDGFR-β) (Abcam) and CD144 antibodies (Thermo) which was used to label vascular endothelial (VE)-cadherin depending on the investigation. Images were viewed under a microscope and acquired and processed using.

### Statistical Analysis

Data were processed using a one-way analysis of variance. A P value < 0.05 was considered statistically significant. Data were displayed as the mean ± standard deviation (SD).

## Results

### The Effects of OCR on HCT116 Cells

**Figure 2:**
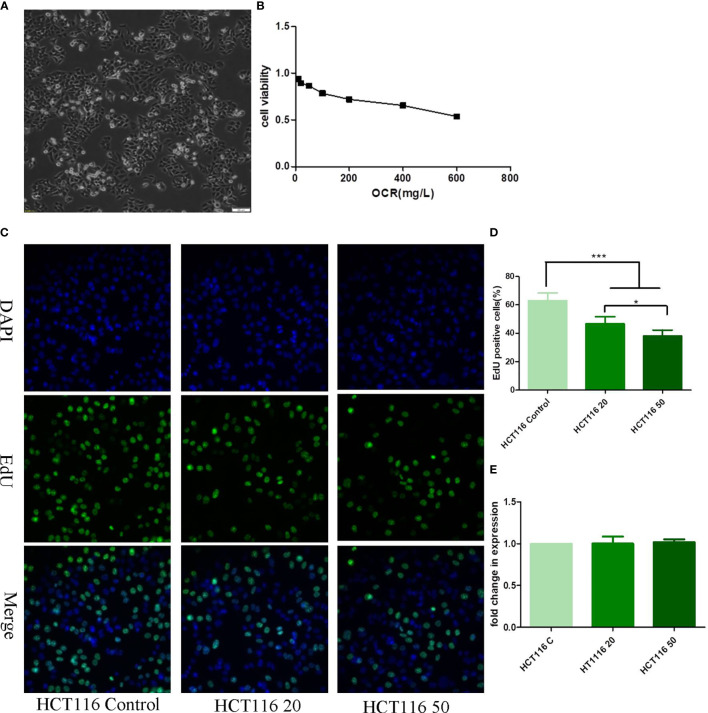
The effects of essential oil from *Curcumae rhizoma* (CR) (OCR) on HCT116 cells. **(A)** HCT116 cell morphology. **(B)** The effects of different OCR concentrations on HCT116 viability. **(C, D)** The effects of OCR on HCT116 proliferation. **(E)** The effects of OCR on VEGFa mRNA expression in HCT116 cells. (n = 3, mean ± standard deviation (SD), ***P < 0.001, *P < 0.05 compared with the control group).

Different OCR concentrations were incubated with HCT116 cells for a 24 h period and cell viability assessed using the CCK-8 method. HCT116 viability decreased with increasing OCR concentrations. HCT116 cells were less tolerant to OCR at IC_50_ = 678.5 μg/ml (**Figures 2A, B**). Based on the OCR intervention curve, two safe doses were selected for drug intervention follow-up studies; a low concentration dose of 20 μg/ml and a medium concentration dose of 50 μg/ml.

To observe the effects of OCR on HCT116 proliferation, we pre-treated the cells with low (20 μg/ml: HCT116 20 group) and medium OCR concentrations (50 μg/ml: HCT116 50 group) for 24 h, and the EdU assay performed to assess proliferation. EdU staining showed that the ratio of EdU-labelled cells at 24 h for both experimental groups was significantly lower than the HT116 control group (P < 0.001). In addition, the ratio of EdU-labelled cells in the HCT116 50 group at 24 h was significantly lower than the HCT116 20 group (P < 0.05) (**Figures 2C, D**). These data suggested that the inhibitory effects of OCR in HCT116 cells were concentration-dependent.

To observe the effects of OCR on VEGFa mRNA expression in HCT116 cells, we pre-treated the cells with low and medium OCR concentrations for 24 h, after which VEGFa mRNA expression was quantified. We observed no significant differences in expression between groups, including the control group (**Figure 2E**).

### The Effects of OCR in HUVECs

**Figure 3:**
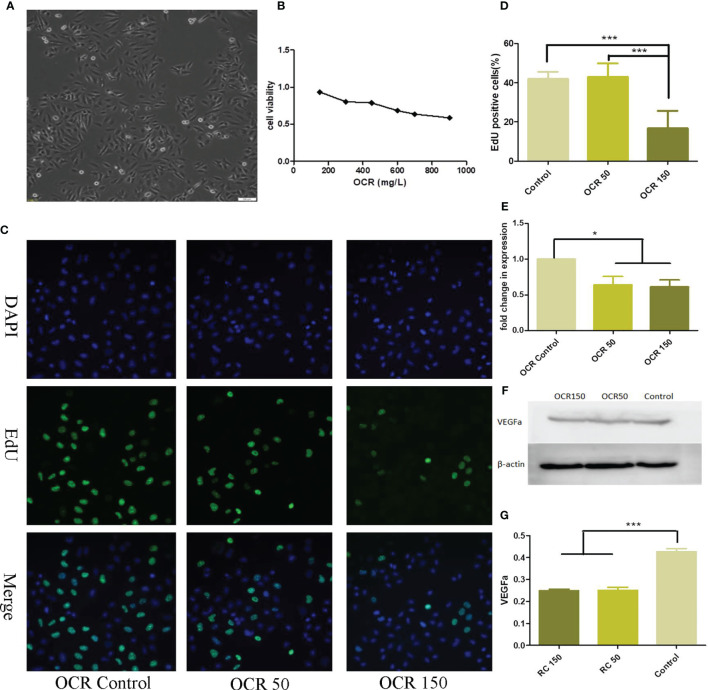
The effects of essential oil from *Curcumae rhizoma* (CR) (OCR) on HUVECs. **(A)** HUVEC morphology. **(B)** The effects of different OCR concentrations on HUVEC viability. **(C, D)** The effects of OCR on HUVEC proliferation. **(E)** The effects of OCR on VEGFa mRNA expression in HUVECs. **(F, G)** The effects of OCR on VEGFa protein expression in HUVECs. (n = 3, mean ± standard deviation (SD), ***P < 0.001, *P < 0.05 compared with the control group).

Different OCR concentrations were incubated with HUVECs for a 24 h period and cell viability assessed using the CCK-8 method. HUVEC viability decreased with increasing OCR concentration. When compared with HCT116 cells, HUVECs were more resistant to OCR, with an IC_50_ = 950μg/ml (**Figures 3A, B**). Based on the OCR intervention curve, two relatively safe doses were selected for drug intervention follow-up studies with HUVECs; a low concentration dose of 50 μg/ml and a medium concentration of 150 μg/ml.

To observe the effects of OCR on HUVEC proliferation, we pre-treated the cells with low (50 μg/ml: OCR 50 group) and medium OCR concentrations (150 μg/ml: OCR 150 group) for 24 h. EdU staining showed that the ratio of EdU-labelled cells at 24 h in the OCR 50 group was not significantly different to the control group. However, the ratio of EdU cells in the OCR 150 group was significantly lower than both control (P < 0.001) and OCR50 groups (P < 0.001) (**Figures 3C, D**).

To determine the effects of OCR on VEGFa RNA expression in HUVECs, we pre-treated the cells with low and medium OCR concentrations for 24 h, after which expression was quantified. VEGFa mRNA expression in both experimental groups were significantly lower than the control group (P < 0.05) (**Figure 3E**).

To observe the effects of OCR on VEGFa protein expression, HUVECs were pre-treated with low and medium OCR concentrations for 24 h, after which western blotting was performed. VEGFa protein expression in both experimental groups was significantly lower than the control group (P < 0.05) (**Figures 3F, G**).

### The Effects of OCR on HUVECs Co-Cultured With HCT116 Cells

**Figure 4:**
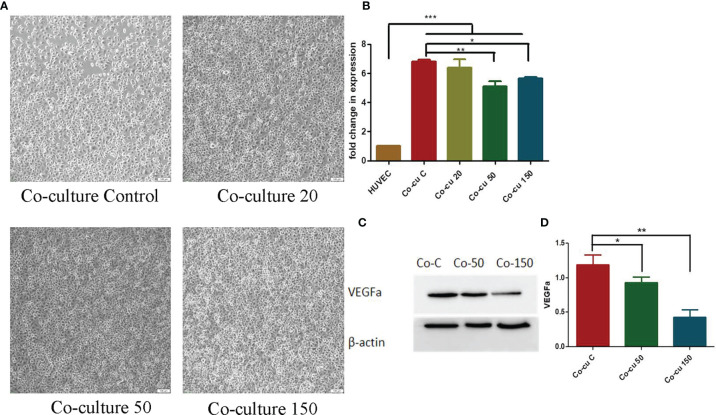
The effects of essential oil from *Curcumae rhizoma* (CR) (OCR) on HUVECs co-cultured with HCT116 cells. **(A)** Changes in HUVEC morphology in the co-culture system at different OCR concentrations. **(B)** The effects of OCR on VEGFa mRNA expression in HUVECs. **(C, D)** The effects of OCR on VEGFa protein expression in HUVECs. (n = 3, mean ± standard deviation (SD), ***P < 0.001, **P < 0.01, *P < 0.05 compared with the control group).

To observe the effects of OCR on HUVEC morphology in a co-culture system, we incubated different OCR concentrations (20 μg/ml, 50 μg/ml and 150 μg/ml) with HUVECs in a co-culture system for 24 h, after which cell morphology was investigated. HUVECs in the co-cultured control group exhibited an irregular cell morphology, longer cell antennae and poor general cell status. In contrast, HUVEC morphology in the co-culture system plus OCR supplementation was relatively regular and cells were in good general condition (**Figure 4A**).

To quantify the effects of OCR on VEGFa mRNA expression in HUVECs in the co-culture system, we pre-treated the system with different OCR concentrations for 24 h and tested. VEGFa mRNA expression in HUVECs in the co-culture system increased significantly compared with HUVECs cultured alone (P < 0.001). When compared with the co-culture control group (co-culture with no OCR treatment), the OCR 50 μg/mland 150 μg/ml intervention groups significantly reduced VEGFa mRNA expression in the co-culture system (P < 0.01, P < 0.05). These data suggested that co-culturing with HCT116 cells increased VEGFa mRNA expression in HUVECs, and the intervention of an OCR dose (50 μg/ml and/or 150 μg/ml) reduced this expression (**Figure 4B**).

To further investigate the effects of OCR on VEGFa protein expression in HUVECs in the co-culture system, the system was pre-treated with different OCR concentrations for 24 h. Western blotting showed that VEGFa protein expression in co-cultured 50 μg/ml and 150 μg/ml OCR groups was significantly lower than the co-cultured control group (P < 0.001). In addition, VEGFa protein expression in the 150 μg/ml co-culture group was significantly lower than the 50 μg/ml co-culture group (P < 0.001) (**Figures 4C, D**). These effects were concentration-dependent.

### The Inhibitory Effects of OCR on Subcutaneously Transplanted Colon Cancer Tumours

**Figure 5:**
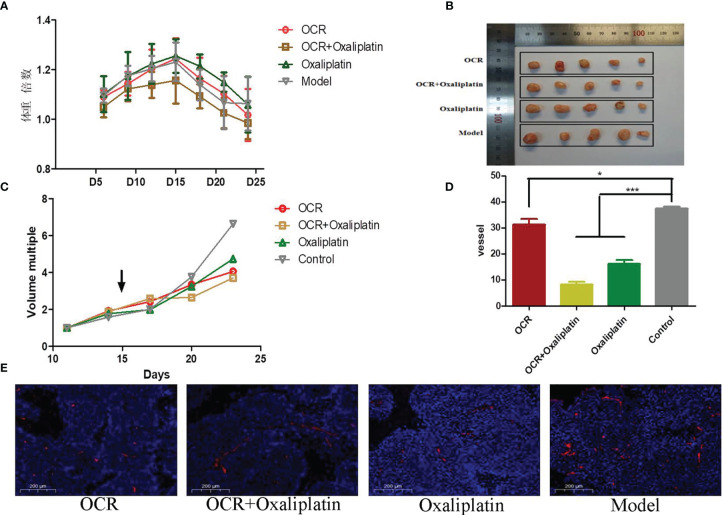
The inhibitory effects of essential oil from *Curcumae rhizoma* (CR) (OCR) on subcutaneously transplanted colon cancer tumours. **(A)** Changes in mice body weight in different groups. **(B, C)** Tumour size in different groups. **(D, E)** CD31 marker expression in tumours in different groups. (n = 5, mean ± standard deviation (SD), ***P < 0.001, *P < 0.05 compared with the control group).

We investigated the effects of OCR on the general state and weight of nude mice. After cells were subcutaneously transplanted, mouse weight was recorded every 3 days. The ratio of body weight to initial body weight after transplantation was taken as the weight multiple of the nude mice. Weight multiple results showed that the weight of nude mice in each group increased variably within 15 days of subcutaneous transplantation; the weight of nude mice in each group decreased to different degrees after 15 days. We observed no differences in this trend between groups, which may have been related to tumour growth (**Figure 5A**).

As specified, tumour growth was recorded every 3 days after transplantation. Tumour volume change data for each group suggested no significant differences between groups within 15 days of transplantation. After OCR administration, tumour growth appeared to differentiate between groups (**Figures 5B, C**). We observed that tumour volume in OCR and combination groups increased slowly.

### The Effects of OCR Blood Vessel Normalisation in Tumours

When compared with the model group, the number of microangiogenesis vessels which was labelled by CD31 in the other groups was significantly reduced (P < 0.001). Also, the number of microangiogenesis vessels in tumours in the OCR + oxaliplatin group were significantly lower than OCR and oxaliplatin groups (P < 0.05) (**Figures 5D, E**). These results suggested that both OCR and oxaliplatin inhibited microangiogenesis vessel formation in tumours, whereas combined use exerted better inhibitory effects in tumour vessels.

**Figure 6:**
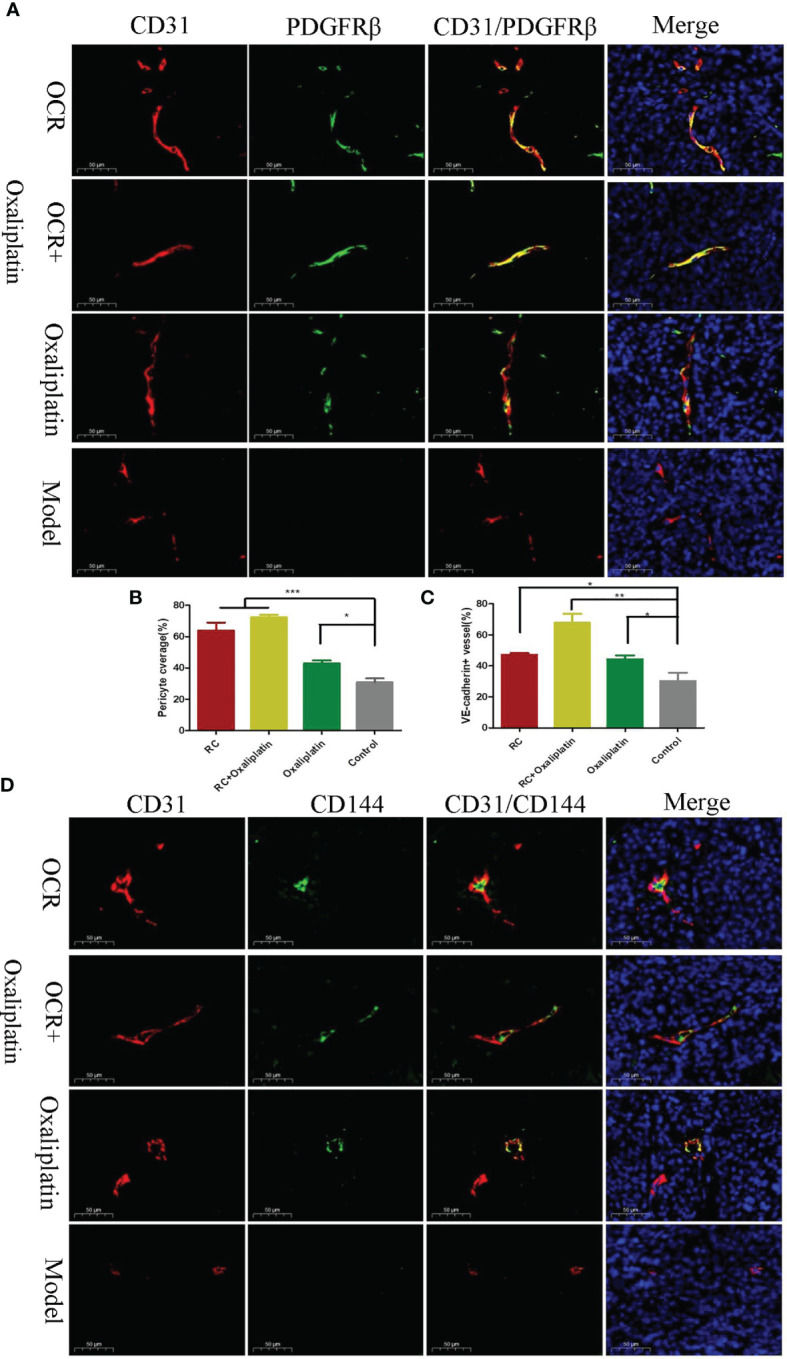
The effects of essential oil from *Curcumae rhizoma* (CR) (OCR) on tumour blood vessel normalisation. **(A, B)** Tumour pericyte coverage in different groups. **(C, D)** VE-cadherin tumour coverage in different groups. (n = 5, mean ± standard deviation (SD), ***P < 0.001, **P < 0.01, *P < 0.05 compared with the control group).

Compared with the model group, PDGFR positive rates in tumour vessels in the other groups significantly increased (P < 0.001). In addition, PDGFR positive rates in the OCR + oxaliplatin group were significantly higher than individual oxaliplatin groups (P < 0.05) (**Figures 6A, B**). These results suggested that both OCR and oxaliplatin increased PDGFR coverage in tumour perivascular cells.

Compared with the model group, the positive rates for vascular endothelial VE-cadherin in tumour vessels of the other groups were significantly increased (**Figures 6C, D**). Also, the positive rate for VE-cadherin in the OCR + oxaliplatin group was significantly higher than the individual OCR and oxaliplatin groups. These results suggested that when combined, OCR and oxaliplatin increased VE-cadherin coverage in tumour vessels.

## Discussion

Previous studies have shown that OCR exerts anti-cancer, anti-inflammatory, liver protection, immune regulation and chemoprevention effects (17). However, no systematic research has yet shown the effects of OCR on tumour vessel normalisation. Therefore, we explored whether OCR inhibited tumour growth by regulating tumour vessels.

OCR elicited inhibitory effects on HUVEC and HCT116 proliferation, with the effects in HCT116 cells concentration-dependent. Also, when compared with HCT116 cells, HUVECs were more resistant to OCR. This tolerance difference between normal and disease model cells suggested OCR exerted a unique intervention effect on diseases. Equally, drug dosage is a great concern for clinicians; we identified that OCR dosage differences in inhibiting normal cell activity and disease model cell activity was a distinct OCR advantage. Thus, when selecting reasonable therapeutic dosages, clinicians can theoretically protect normal cells while inhibiting diseased cells.

VEGFa is an important angiogenic factor. Its vascular permeability allows macromolecular proteins such as fibrinogen to leak from blood vessels, thereby forming capillary blood networks (5). Also, VEGFa selectively acts on vascular endothelial cells to promote proliferation and migration, to ultimately form new blood vessels (21). In the tumour environment, massive tumour metabolic consumption, severe local tumour hypoxia and large metabolite accumulation feeds back to stimulate tumour cell secretion of a variety of angiogenesis-related factors, thereby rapidly forming new blood vessels. VEGFa is one such fast-response angiogenic factor (22). Studies have confirmed VEGFa is highly expressed in various tumours in the liver, breast, colon and melanoma cancer (23–27). We observed that OCR inhibited VEGF RNA and protein expression in HUVECs. When co-cultured with colon cancer cells, HUVECs displayed abnormal morphology. Also, VEGFa expression in HUVECs in the disease co-culture model was significantly higher than in HUVECs cultured alone.

These data verified the traditional tumour vessel theory to a certain extent. During HUVEC co-cultivation with HCT116 cells, these cells secreted molecules that acted on HUVECs, thereby facilitating a significant increase in VEGFa mRNA expression. This observation indicated that the co-culture system accurately reflected changes in endothelial cells during tumour pathology. Our data also suggested that an OCR intervention (50 μg/ml and 150 μg/ml) reversed this rapid increase in VEGFa RNA expression in HUVECs. Western blotting evidence also suggested that OCR (50 μg/ml and 150 μg/ml) treatments significantly reduced VEGFa protein expression in HUVECs cultured alone and co-cultured HUVECs. Importantly, these inhibitory effects were concentration-dependent. Therefore, these *in vitro* investigations suggested that OCR may inhibit endothelial cells’ overgrowth during cancer pathological conditions.

Many factors in the body promote angiogenesis, with compensatory mechanisms operating under certain conditions (28). Currently, no single characteristic factor reflects the maturity of tumour vessels (29). Therefore, to further dissect the effects of OCR on tumour vessels and closely observe these effects on tumour vessel morphology and structure, we conducted comprehensive *in vivo* studies. Our weight results in mice showed that OCR combined with oxaliplatin had no significant effects on these data. Also, tumour volume changes in mice showed that individual OCR and combined with oxaliplatin slowed down tumour growth rates.

According to tumour vessel characteristics, reducing tumour angiogenesis rates and improving vessel structure are effective strategies in normalising tumour vessels. Therefore, we explored the impact of OCR on blood vessels in solid tumours. Platelet endothelial cell adhesion molecule-1 (CD31) is located at tight junctions between vascular endothelial cells and may be used to assess tumour angiogenesis. Studies have shown CD31 is expressed in both mature and immature tumour vessels. When compared with other endothelial cell marker factors, CD31 more accurately reflected the number and density of tumour microvessels (30). Our CD31 data indicated that the number of microangiogenesis vessels in the model group was higher than other groups. This was consistent with the literature where blood vessels are rapidly and disorderly formed in tumours. In our work, OCR reduced the numbers of microangiogenesis vessels in tumours, whereas the combination with oxaliplatin increased them. In addition, this combination altered disordered tumour microvessels to continuous and orderly vessels.

Pericytes are important components of arterioles, veins, capillaries (31), providing support for endothelial cells (32). Within the vascular structure, endothelial cells surround to form a lumen. Pericytes adhere to endothelial cells and the basement membrane which surrounds them (33). Thus, pericytes cover endothelial cells and help quiescent endothelial cells maintain a non-proliferative state. However, during tumour growth, pericytes lose their monitoring capabilities which cause endothelial cells to become activated in the presence of stimulating angiogenic factors (34). Studies have shown that it is vital to restore pericyte coverage in the vasculature in a tumour state (35). From our study, OCR appeared to restore perivascular cell coverage in the tumour, and similarly, OCR and oxaliplatin combined increased this effect.

VE-cadherin is an endothelial cell membrane protein. Physiologically, it regulates contact inhibition between vascular endothelial cells (36). VE-cadherin also generates a dynamic balance between endothelial cell growth and perictyte coverage in blood vessels and helps maintain blood vessel homeostasis. Also, VE-cadherin regulates the growth rate of vascular endothelial cells by regulating angiogenic factors (37, 38). Studies have shown that *VE-cadherin* knockout mice experience difficulties forming initial continuous blood vessels due to adhesion barriers between endothelial cells, causing death within 10 days of embryonic development (39). Similarly, in *VE-cadherin* knockout newborn mice, new endothelial cells cannot connect to form new blood vessels, whereas existing endothelial cells in original blood vessels were released (40). Under normal physiological conditions, VE-cadherin is tightly connected to endothelial cells so that cells are ordered and structurally complete. When VE-cadherin expression is lacking (knocked out), endothelial cells cannot undergo monolayer formation and arrangement. In our study, OCR improved VE-cadherin coverage in tumours, and similarly, when combined with oxaliplatin, these effects were enhanced. Thus, OCR inhibited tumour growth, reduced tumour angiogenesis and improved abnormal vascular structures in mouse tumours.

## Conclusions

**Figure 7:**
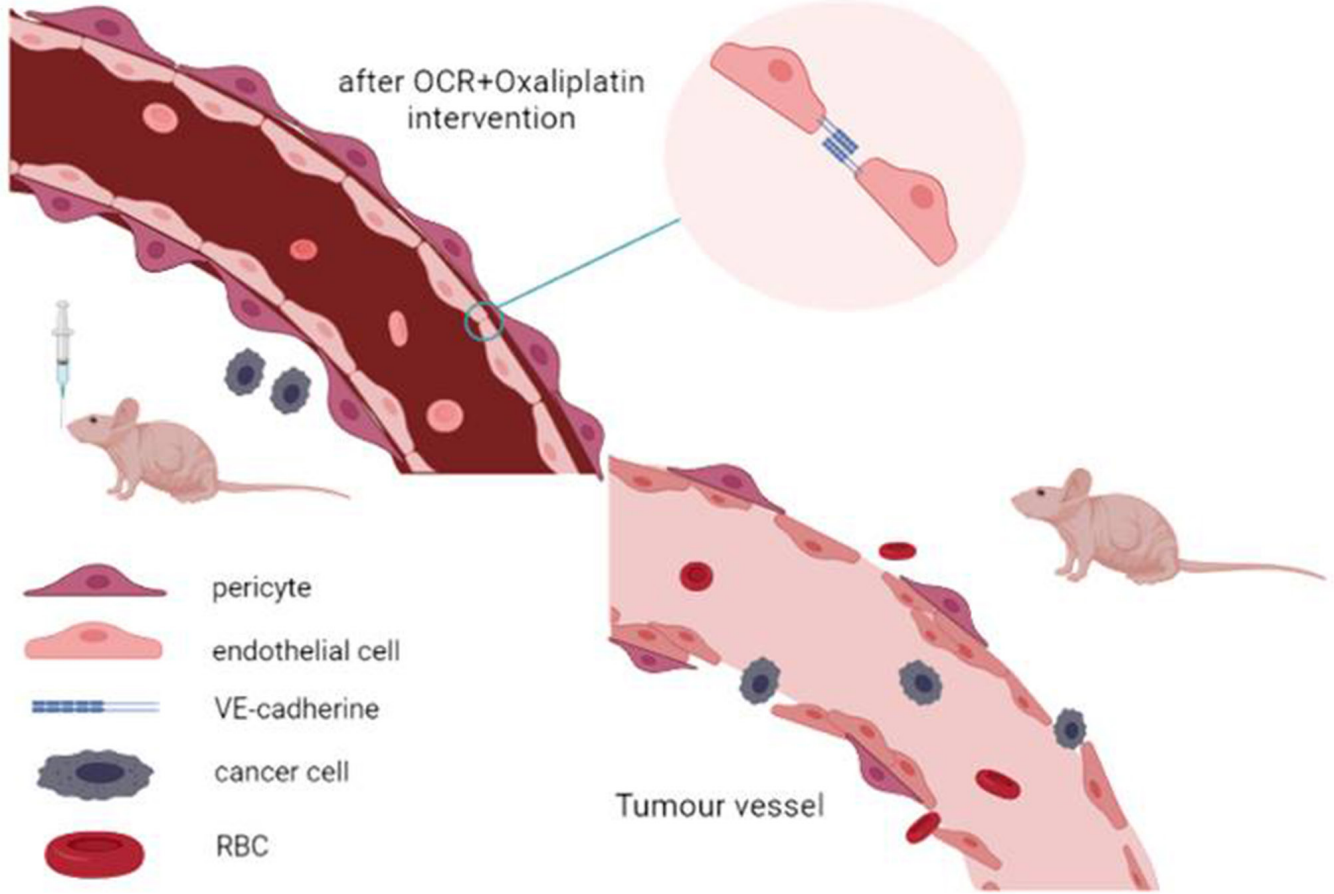
Conclusion diagram.

OCR inhibited HCT116 and HUVEC activity, proliferation and also VEGFA mRNA and protein expression in HUVECs. It reversed VEGFA mRNA and protein overexpression in HUVEC cells under pathological condition. OCR also inhibited subcutaneously transplanted colon cancer tumour growth rates in mice. Finally, OCR combined with oxaliplatin reduced tumour angiogenesis and increased VE-cadherin and pericyte coverage, thus normalising tumour vascular structure (**Figure 7**).

## Data Availability Statement

The raw data supporting the conclusions of this article will be made available by the authors, without undue reservation.

## Ethics Statement

The animal study was reviewed and approved by Animal Ethics Committee of Chengdu University of Traditional Chinese Medicine (Animal Ethics Approval Number: 2017-08).

## Author Contributions

YF: Validation, methodology, and writing-original draft. LD: Methodology. HG: Methodology. YZ: Validation. FP: Conceptualization, writing-reviewing. and editing. GW: Writing-reviewing and editing ideas. CY: Conceptualization, writing- reviewing and editing, and project administration. All authors contributed to the article and approved the submitted version.

## Funding

This work was supported by the National Natural Science Foundation of China (No.81673878), Experimental Formulary Sichuan Youth Science and technology Innovation research team (2020JDTD0022), and Youth Innovation Promotion Association of the Chinese Academy of Sciences (CACM-2020-QNRC1-01).

## Conflict of Interest

The authors declare that the research was conducted in the absence of any commercial or financial relationships that could be construed as a potential conflict of interest.

## Publisher’s Note

All claims expressed in this article are solely those of the authors and do not necessarily represent those of their affiliated organizations, or those of the publisher, the editors and the reviewers. Any product that may be evaluated in this article, or claim that may be made by its manufacturer, is not guaranteed or endorsed by the publisher.

## Abbreviations

CR, *Curcumae Rhizoma*; OCR, essential oil from CR; HUVEC, Human umbilical vein endothelial cells; VEGFa, Vascular endothelial growth factor-a.
